# F30. SMARTPHONE APPLICATION “ROBIN”: FEASIBILITY, ENGAGEMENT AND SATISFACTION OF A SMARTPHONE APPLICATION APPROACH TO SUPPORT TREATMENT OF (ATTENUATED) PSYCHOTIC SYMPTOMS IN ADOLESCENTS

**DOI:** 10.1093/schbul/sby017.561

**Published:** 2018-04-01

**Authors:** Nina Traber-Walker, Sibylle Metzler, Miriam Gerstenberg, Susanne Walitza, Maurizia Franscini

**Affiliations:** University Hospital Zurich

## Abstract

**Background:**

There is increasing interest in using mobile technologies such as smartphones application in mental health care. First research results from the use of smartphone applications in the treatment of psychotic disorders are promising. Current analysis showed, that especially young people would be interested in smartphone applications within treatment settings. However, there is a lack of investigations in this population. There is also little known about mobile technologies in the work with attenuated psychotic symptoms. To address these gaps, we developed “Robin”, a specific smartphone application to support the therapy of adolescents with attenuated or full-blown psychotic symptoms. The smartphone application targets medication adherence, real-time symptom assessment and provides help coping with symptoms and stressful situations in daily life.

**Methods:**

Based on existing literature and our clinical expertise within a specialized outpatient care for adolescents with (attenuated) psychotic symptoms, a first modular version of the app was developed and adapted after first pilot investigations with patients (N=7, Age 14–18) and therapists (N=10). Participants of the pilot investigation completed a questionnaire regarding usability and acceptance of the application. Furthermore, we investigated how the patients used the application in their daily life by analyzing the user data from the application. In September 2017, the development of the smartphone application has been finalized and we have started with a systematic clinical evaluation study for testing the efficiency of the app. The application is only used in combination with psychotherapy in our university hospital for child and adolescent psychiatry.

**Results:**

The data from our pilot investigation showed, that “Robin” was accepted by clinicians and patients. All clinicians said they would like to use the application to enrich their therapeutic approaches. All patients in the pilot project used the application in their daily life. Especially modules with information about symptoms and coping strategies were frequently used. Since September 2017, first patients have been included to the systematic evaluation study. In Florence 2018, we will present first data from this study about feasibility, engagement and subjectively perceived benefit of the smartphone application.

**Discussion:**

The first feedbacks from the pilot investigation were encouraging. The findings were used to improve and adapt the application. Since September 2017, the application is used in psychotherapy and an evaluation study has started. This is one of the first clinical trials to test the efficacy of a specific application developed for adolescents with psychotic and with attenuated psychotic symptoms.

